# New approach for hepatocellular carcinoma treatment

**DOI:** 10.25122/jml-2021-0088

**Published:** 2022-01

**Authors:** Daniela Tabacelia, Cezar Stroescu, Radu Dumitru, Raluca Roxana Grigorescu, Alexandru Martiniuc, Ioana Alexandra Husar-Sburlan, Narcis Copca

**Affiliations:** 1. Department of Gastroenterology, Sfanta Maria Clinical Hospital, Bucharest, Romania; 2. Department of Surgery, Sfanta Maria Clinical Hospital, Bucharest, Romania; 3. Department of Surgery, Fundeni Clinical Institute, Bucharest, Romania; 4. Radiology Department, Sfanta Maria Clinical Hospital, Bucharest, Romania; 5. Radiology Department, Fundeni Clinical Institute, Bucharest, Romania

**Keywords:** hepatocellular carcinoma, microwave ablation, transarterial chemoembolization, HCC – hepatocellular carcinoma, MWA – microwave ablation, TACE – transarterial chemoembolization, RFA – radiofrequency ablation, PEI – percutaneous alcohol injection, AFP – alpha-fetoprotein

## Abstract

Hepatocellular carcinoma (HCC) is the fifth most common cancer, with an increasing incidence in recent years. The prognosis is unfavorable, representing the third most frequent cause of cancer-related death worldwide. This is because it generally develops in patients with pre-existing liver pathology, thus limiting therapeutic options. The role of ablative therapies is well-established in nodules smaller than 3 cm, but for nodules from 3 to 5 cm, the best therapeutic management is not well defined. Recent studies reported that combining minimally invasive procedures like transarterial chemoembolization (TACE) with microwave ablation (MWA) or radiofrequency ablation is superior to each alone. However, there is no consensus regarding the timing and the order in which each procedure should be performed. We report a case of an 86 years old male with HCV-related compensated hepatic cirrhosis and multiple cardiac comorbidities diagnosed with a 47/50 mm HCC. Pre-surgical evaluation of the associated pathologies determined that the risk for the surgical approach outweighs the benefits, so the committee decided to treat it in a less invasive manner. We performed MWA and TACE in a single session with technical success according to the modified Response Evaluation Criteria in Solid Tumors (m-RECIST). This case illustrates the first case of simultaneous MWA and TACE performed in our center. This new approach of hepatocellular carcinoma appears to be a good alternative to more invasive methods, with good results even in older people that are unfit for surgery.

## Introduction

Hepatocellular carcinoma (HCC) is the third cause of cancer-related death worldwide [1]. The increased mortality is given because the treatment should consider four related aspects: tumor stage, performance status of the patient, treatment efficacy, and the death cause of cirrhotic patients with HCC, including decompensated cirrhosis, metastases of HCC, and rupture of HCC with hemoperitoneum [2–5]. Liver transplantation is the best option for patients with HCC and liver cirrhosis that are fit according to Milan criteria [6]. However, less than 20% of patients with HCC benefit from either resection or transplantation due to tumor characteristics or decompensated cirrhosis, and for them, the last treatment option remains interventional or palliative therapies [7].

The interventional therapies available are transarterial chemoembolization (TACE), radiofrequency ablation (RFA), microwave ablation (MWA), and percutaneous alcohol injection (PEI). Although these procedures are safe and effective for small tumors, the effectiveness decreases for tumors larger than 3 cm, which is why more recent studies describe the advantages of combination therapy between two or three of these procedures [8–10].

## Case Report

**Figure 1.**
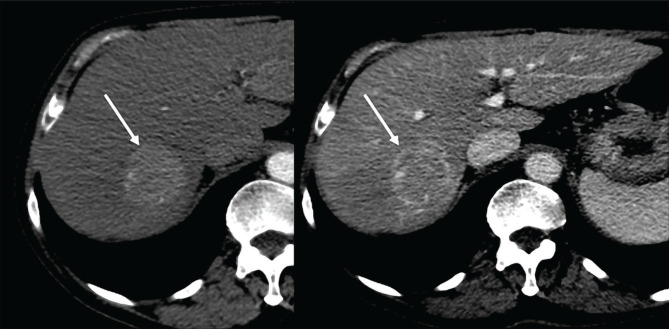
Enhanced CT, arterial and portal phase. A large hepatic nodule (5 cm) is seen in the VIIth segment, with arterial enhancement and rapid washout in portal phase (arrow), a typical CT appearance of HCC.

**Figure 2.**
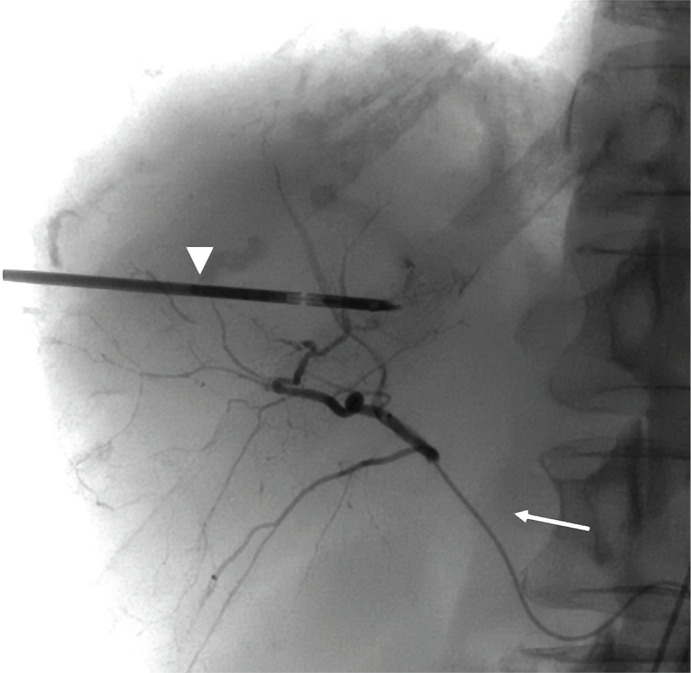
An MW probe (arrowhead) is placed under US guidance. A microcatheter (arrow) is placed in the posterior right hepatic artery for the deployment of DEB in the TACE procedure.

**Figure 3.**
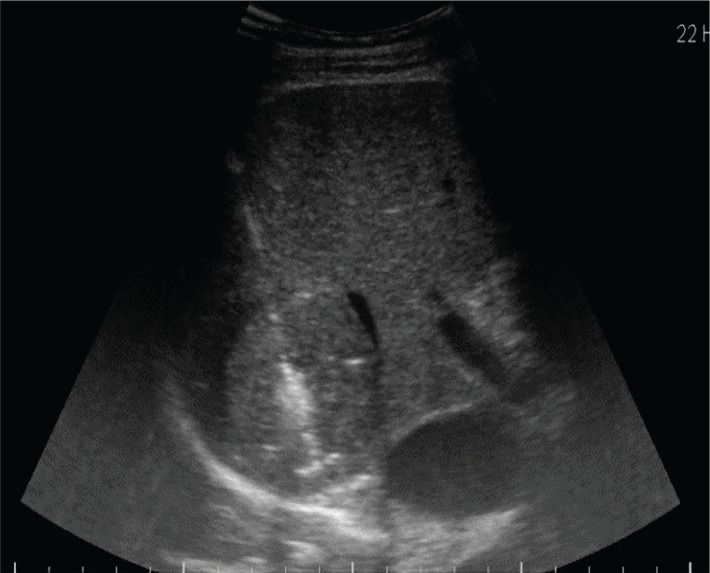
MW ablation probe placement in the nodule during ablation.

We present a case of an 86-year-old man, known with arterial hypertension, ischemic cardiomyopathy, sinus bradycardia (47–55 b/min), left bundle branch block, compensated cirrhosis due to chronic hepatitis C virus (HCV) infection, diagnosed at a CT scan with a 47/50 mm hepatocellular carcinoma in the 7^th^ liver segment (Figure 1) who was referred to our hospital for resection. The preliminary blood test revealed mild thrombocytopenia, elevated liver enzymes, increased serum alpha-fetoprotein (AFP) 48.07 IU/ml (normal range 0.5–5.5), and CA 19-9 153.87 U/ml (normal range 0–33 U/ml). Superior digestive endoscopy showed small esophageal varices without high-risk stigmata. Corroborating paraclinical data, the patient was classified as Child-Pugh A (5 pt) cirrhosis, with an HCC according to the Barcelona Clinic liver cancer (BCLC) staging system, also stage A. The case was discussed in the multidisciplinary board, but due to multiple cardiovascular comorbidities, the patient had an increased surgical risk, and minimally invasive combined therapy TACE and MWA with curative intent were decided. The procedures were done under general anesthesia with endotracheal intubation, with the patient in the supine position, in the angiographic department. We performed tumor ablation first using the Medtronic Emprint system. The tumor was identified by ultrasound and, after preparing the puncture site in a sterile manner and applying the local anesthetic, the nodule was punctured using a 14 G evident antenna with a water circulation cooling system. Next, we punctured the tumor with a 17 cm long antenna with an active tip of 3.7 cm under ultrasound guidance (Figure 2). We set the microwave power delivered at 45 watts at 2.45 GHz for 10 minutes (Figure 3), and the hyperechoic spots around the antenna appeared, confirming that the heat was applied. During the retraction of the antenna, we continued the ablation to achieve needle track ablation.

**Figure 4.**
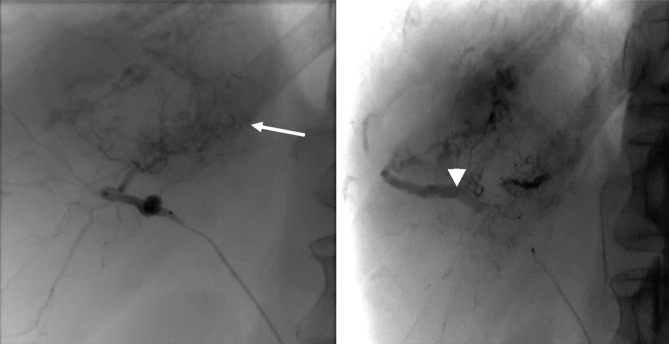
After the ablation, a large area of lack of enhancement is seen in ¾ of the tumor, with residual enhancement on the medial side (arrow) for which DEB-TACE is performed immediately. Large arterioportal shunt (arrowhead) is seen in the dynamic acquisition.

**Figure 5.**
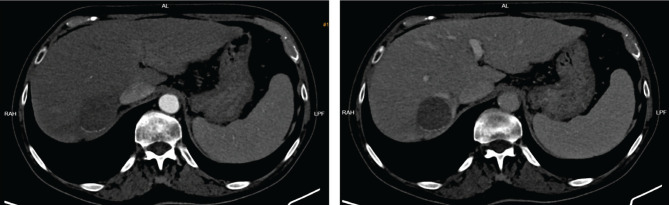
CT scan with intravenous contrast at 12 months post-procedure (arterial and venous phase) – complete tumor necrosis.

Immediately afterward, TACE was performed. Following skin sterilization, the catheter was introduced through the femoral artery using Seldinger’s technique. An angiogram was performed to visualize the tumor supplying artery. Subsequently, using a microcatheter (Terumo Progreat 2,8F), we achieved super-selective cannulation of the feeding artery. Angiography showed the absence of tumoral blush in the part of the tumor treated by MWA and the appearance of a large arterio-portal shunt, which was bypassed using the microcatheter (Figure 4). We performed TACE with drug-eluting beads (DEBs- Boston Scientific, Tandem 100 microns), charged with 100 mg Doxorubicin until complete stasis was achieved in the tumoral feeder. At 24 hours after the procedure, a slight elevation of transaminases was seen. The follow-up program was 18 months, and it included abdominal enhanced CT/MRI at one month and then every three months after tumor ablation to evaluate the treatment effectiveness and observe if any residual viable tumor remained. Tumor response was assessed according to the modified Response Evaluation Criteria in Solid Tumors (m-RECIST) [11], and complete response (CR) and normalization of AFP values were observed every time during follow-up (Figure 5).

## Discussion

Using BCLC staging, our patient was placed in stage A, but because he was declared unfit for surgery due to his comorbidities, a minimally invasive approach with curative intent seemed a good option. Effective minimally invasive treatment is considered when tumor destruction occurs with 10 mm margins – the equivalent of surgical resection, without causing damage to healthy surrounding tissue or other non-targeted structures [12]. Although TACE is the recommended locoregional therapy, the recurrence rate after this procedure alone is high [13]. Similarly, for thermal ablation, it was observed that recurrent tumors could occur in a distant liver segment from the treated HCC or disseminated to more than one liver segment [14, 15]. Therefore, association with TACE can control microlesions and increase the probability of complete necrosis of satellite nodules, improving patient outcomes [16].

In the last years, researchers have shown better response and higher overall survival after combination therapy for early and intermediate tumors [17]. The vast majority of studies that compare TACE and RFA show that using them together leads to a better outcome than used separately [18, 19]. In contrast to RFA, microwave ablation is a relatively new thermal procedure that causes oscillation of polar water molecules at high frequency generating kinetic energy and frictional heating over 1000 C, causing cellular death by desiccation and char [20, 21]. MWA procedure has several advantages, such as faster heating over a higher volume, lower susceptibility to local perfusion, and attenuated sink-effect, which translate into a shorter procedure time, predictable volume ablation, and the possibility to treat larger tumors and create larger ablation areas in less time than RFA [22].

The complication rates reported after MWA are relatively low, 2.6–2.9% [23, 24], and include bile duct injuries, hemorrhage, liver abscess, colon perforation, skin burns, tumor seeding, and hemoglobinuria [25]. Although bleeding in rare cases requires intervention, performing TACE immediately after MWA can treat this complication. Another complication that can occur after MWA that can be treated with TACE is the appearance of arteriovenous fistula [26]. Studies that compare different combined treatments applied in a single session, with the ablation performed before TACE, found that MWA with TACE is more effective than RFA and TACE and has the same complication rate as RFA, but less than TACE alone [27]. There is no consensus regarding the timing and order in which MWA and TACE may be performed [28, 29]. The advantage of performing MWA after TACE is decreased local blood flow that minimizes heat loss through convection by increasing the thermal effect of the ablation [26], while the chemotherapy agent mixed with lipiodol increases the sensitivity of tumor cells to temperature, thus increasing the effectiveness of the ablation [30]. The advantage of TACE after thermal ablation is that the drugs used in TACE enhance the necrosis induced by MWA [26]. In our case, we decided to combine TACE with MWA. We began with MWA because the sink effect during this procedure is relatively low, and we considered that the risk of new arteriovenous shunts’ appearance is higher.

Immediately after microwave ablation, angiography showed the creation of a large arterio-portal shunt, which could be embolized by DEB-TACE, thus reducing the risk of liver metastases. In addition, the angiographic control showed complete tumoral destruction, and the follow-up CT scans did not show recurrence over 18 months. In our case, the patient did not develop any vascular, septic, or biliary complications but showed a slight elevation in transaminases that normalized in a few days.

## Conclusion

This case report highlights the technical considerations and indications of combining different minimally invasive procedures in treating an HCC nodule in a cirrhotic patient with curative intent and the importance of managing these patients in centers where all these techniques are readily available. Percutaneous MWA combined with TACE in the same session seems to be a safe and effective alternative to liver resection in selected cases, but more randomized trials are necessary to confirm this data and develop a consensus regarding the order and the best timing for each procedure.

## Acknowledgments

### Conflict of interest

The authors declare no conflict of interest.

### Consent for publication

All patients admitted to our hospital signed an informed consent in which they stated that they understood that the information or pictures related to their illness might be part of a study, published without a name attached.

### Personal thanks

We thank Popa Laura and Florescu Madalina for their expertise and assistance throughout all aspects of our study and for their help in writing the manuscript.

### Authorship

DT, CS, RD contributed to conceptualizing, methodology, and writing the original draft. RG, AM, IH contributed to data collection, data analysis, and editing of the manuscript. NC contributed to the editing and review of the manuscript.
